# How to make use of unlabeled observations in species distribution modeling using point process models

**DOI:** 10.1002/ece3.7411

**Published:** 2021-04-01

**Authors:** Emy Guilbault, Ian Renner, Michael Mahony, Eric Beh

**Affiliations:** ^1^ Faculty of Science School of Mathematical and Physical Sciences The University of Newcastle Callaghan NSW Australia; ^2^ Faculty of Science School of Environmental and Life Sciences The University of Newcastle Callaghan NSW Australia

**Keywords:** classification, ecological statistics, EM algorithm, machine learning, misidentification, mixture modeling, presence‐only data

## Abstract

1. Species distribution modeling, which allows users to predict the spatial distribution of species with the use of environmental covariates, has become increasingly popular, with many software platforms providing tools to fit such models. However, the species observations used can have varying levels of quality and can have incomplete information, such as uncertain or unknown species identity.
2. In this paper, we develop two algorithms to classify observations with unknown species identities which simultaneously predict several species distributions using spatial point processes. Through simulations, we compare the performance of these algorithms using 7 different initializations to the performance of models fitted using only the observations with known species identity.
3. We show that performance varies with differences in correlation among species distributions, species abundance, and the proportion of observations with unknown species identities. Additionally, some of the methods developed here outperformed the models that did not use the misspecified data. We applied the best‐performing methods to a dataset of three frog species (*Mixophyes*).
4. These models represent a helpful and promising tool for opportunistic surveys where misidentification is possible or for the distribution of species newly separated in their taxonomy.

## Graphical abstract

Data quality in species distribution modeling is a central interest to improve species distribution prediction. We developed two algorithms with 7 initialization methods to classify data with uncertain species identity. We use simulations to test the model parameters and choose the best methods to apply to *Myxophies* genus species.

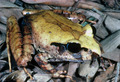

## 1 INTRODUCTION AND BACKGROUND

Species distribution modeling has been a popular topic in ecological statistics over the past decade. Many tools and methods have been developed to provide a means to explore the distributions of species through mapping of suitable environments (Inoue et al., 2017; Jewell et al., 2007; Nezer et al., 2016; Peterman et al., 2013; Schank et al., 2017). Although there are a large number of algorithms and software platforms that can fit species distribution models (SDMs), generalization of these methods and specific applications to real datasets can be tricky (Aarts et al., 2012; Burnham & Anderson, 2002; Guillera‐Arroita et al., 2015).

The most common sources of species information used in SDMs are presence‐only (PO) and presence–absence (PA) data. PO data only contain information about species presences, in contrast to PA data which records both where species have been found present and where they have not been found (Renner et al., 2015; Warton & Shepherd, 2010). Although PA data are generally of higher quality, it is also less common than PO data because it requires more rigorous planning to visit a set of predetermined sites. On the other hand, PO datasets are very common, arising from surveys or opportunistic sightings, but they usually have lower quality (Ruete & Leynaud, 2015; van Strien et al., 2013). Point process models (PPMs) are a common tool for fitting SDMs to analyze PO data (Mi et al., 2014; Renner et al., 2015; Warton & Shepherd, 2010) and have been used to fit models for real datasets and simulated data (Baddeley et al., 2006, 2015; Illian et al., 2012; Renner & Warton, 2013).

Unreliable or unknown species identification is the main concern in ecology especially for PO data from citizen science. Another issue can arise from confounded records when species taxonomy changes (Mahony et al., 2006). For example, *Mixophyes* frogs are now classified in three genetically distinct species while previously only one species was recognized. The *Mixophyes* frogs are not an isolated case. Padial and De la Riva (2006) noted that taxonomy inflation and new species discovery had contributed to an increase of 48.7% in new species of various organisms by that time. In particular, they refer to a study from Köhler et al. (2005) where amphibian species counts had increased by 25% from 1992 to 2004. This increase in reclassified and new species raises challenges to conservation biology (Catenazzi, 2015; Padial & De la Riva, 2006). Conservation planning efforts depend on clear identification of species and understanding of their distributions and habitat requirements (Franklin, 2013; Guisan et al., 2013). Other than cleaning datasets with missing information, little else is typically done in SDMs to account for misspecification. These practices can lead to missing information and thus incomplete predictions. Consequently, there are new challenges in building appropriate species distribution models for such species, for which the *Mixophyes* example serves as an illustration.

One way we can consider dealing with unknown species identities is to relabel them using mixture modeling or machine learning algorithms. Mixture modeling is a common tool used to represent complex distributions and aims to identify different groups within a dataset while modeling heterogeneity (Fernández Martinez, 2015; Hui, 2016). In communities or groups of species, it is possible to classify or cluster species according to covariate information through their preferences by using finite mixture modeling (Dunstan et al., 2013; Fernández‐Michelli et al., 2016; Frame & Jammalamadaka, 2007; McLachlan & Peel, 2000). One particular application of this approach is to deal with over‐dispersed data and to model ecological processes in parallel for different species (Matthews et al., 2001; Tracey et al., 2013; Zhang et al., 2004).

Machine learning algorithms are also becoming more common in statistical ecology because they can make use of unknown information and recognize specific structure in the data (Browning et al., 2018; Hastie et al., 2001; Thessen, 2016). Several algorithms exist such as unsupervised learning algorithms that can group observations with similar characteristics. Supervised learning algorithms use separate labeled datasets for classification and semisupervised learning algorithms learn from partially labeled data within the studied dataset to classify the observations (Fernández‐Michelli et al., 2016; Vo et al., 2018; Wendel et al., 2015; Zhou, 2018). Recent publications have applied machine learning algorithms to fit PPMs in a Bayesian framework (Tran, 2017; Vo et al., 2018), but the literature on using machine learning algorithms to fit PPMs is not yet well developed. Additionally, several R packages apply machine learning procedures for classification procedures (Benaglia et al., 2009; Iovleff, 2018), but none accommodate the intersection of point process models with mixture modeling or machine learning algorithms.

In this paper, we develop new tools for fitting models to multispecies PO data with partial species identification by combining the PPM framework with mixture modeling and machine learning approaches to accommodate incomplete labeling. Our proposed methods rely on classification of points with unknown species labels based on the locations with known species labels. Hence, these methods will only assign classifications of known species in the region with verified species labels. The first tool employs an iterative technique to fit separate PPMs to points with known labels augmented by some points with unknown labels depending on classification probabilities at each iteration. This method will be hereafter known as the *Loop method*. The second tool fits mixtures of PPMs to all available data with an expectation–maximization (EM) algorithm and uses them to classify the unlabeled points. This method will be called *mixture method*. Using simulations, we compare the performance in classification and prediction for the proposed algorithms to the simple, standard approach of fitting individual PPMs to the points with known species labels only. In this article, we will first define the new algorithms developed in Section 2. Then, we describe how we apply these methods to simulated data sets showcasing differences in abundance, correlation between species distributions, and percentage of data with unknown species labels in Section 3, as well as to the *Mixophyes* dataset we previously mentioned in Section 3.3. We present the results of these analyses in Section 4 and provide a discussion in Section 5.

## 2 NEW MODELING METHODS

### 2.1 Background

In ecology, we will consider a spatial point pattern as the distribution of species observation records over a specific window or study area A. The point pattern intensity is defined as the density of points per unit area throughout A. For Poisson point processes, we model the intensity of species *i* as a log‐linear function of covariates **x**:

(1) $$\ln\left(\mu_{i}\left(s\right)\right)=\beta_{i,0}+\sum_{j}x_{j}\left(s\right)\times \beta_{i,j}$$

with $\mu_{i}\left(s\right)$ being the intensity of species *i* at location *s*. Here, *x_j_* contains the values of covariate *j*, with which we associate the parameter *β_i_*_,_ *_j_*. We fit a point process model by maximizing the log‐likelihood, as follows:

(2) $$\ell \left(\beta_{i},s\right)=\sum_{1}^{m_{i}}\text{ln}\left(\mu_{i}\left(s\right)\right)‐\int_{A}\mu \left(s\right)ds$$

here **β** *_i_* is the set of parameters associated with the covariates **x** and **s** is the set of **m_i_** presence locations. The integral is intractable so we rely on numerical quadrature to get an estimate.

### 2.2 Notation

**FIGURE 1:**
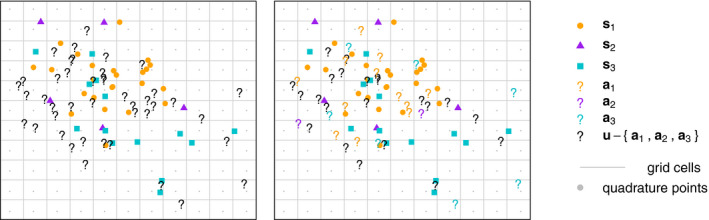
Three illustrative point patterns. The orange dots, purple triangles, and turquoise squares represent locations with known species identity, $s_{1}$, $s_{2}$, and $s_{3}$. The gray dots represent quadrature points q, which are spaced evenly along a regular grid such that one quadrature point is at the center of each rectangular grid cell. The black question marks (left and right) represent observed locations u with uncertain species identity. Some locations in $a_{1}\in u$, $a_{2}\in u$, and $a_{3}\in u$ are classified as belonging to one of the species are represented by colored question marks (right)

The fitted point process models in our proposed methods make use of a total of M+N+Q locations as follows:

Let $s_{1}=\left\{s_{1},\ldots ,s_{m_{1}}\right\}$, $s_{2}=\left\{s_{m_{1}+1},\ldots ,s_{m_{1}+m_{2}}\right\}$, …, $s_{K}=\left\{s_{M‐m_{K}+1},\ldots ,s_{M}\right\}$ be vectors that contain all of the observed locations with known species identities 1,2,…,K, respectively. These are represented by the orange dots, purple triangles, and turquoise squares in Figure 1 for a hypothetical dataset. Let $\left|s_{1}\right|=m_{1},\left|s_{2}\right|=m_{2},\ldots ,\left|s_{K}\right|=m_{K}$ be the number of observed locations with known species identity for each of the *K* species. We collect the $M=m_{1}+m_{2}+\ldots +m_{K}$ total locations with known species identities of all *K* species in $s=\left\{s_{1},s_{2},\ldots ,s_{K}\right\}$. Let $u=\left\{s_{M+1},\ldots ,s_{M+N}\right\}$ contain the *N* observed locations with uncertain species identities. These are represented by the question marks in Figure 1.

Let $q=\left\{s_{M+N+1},\ldots ,s_{M+N+Q}\right\}$ contain the locations of *Q* quadrature points placed along a regular $c_{1}\times c_{2}$ grid throughout the study region (Figure 1). Each quadrature point is placed at the center of one of *Q* unique rectangular grid cells throughout the study region. Let *c*(*s*) be the grid cell in which location *s* is contained.

The proposed Loop and Mixture methods presented in Sections 2.3 and 2.4 assign some of the observations with uncertain species identities in **u** to the set of locations with known species identities in **s**, as in the right panel of Figure 1.

### 2.3 Loop methods

The three‐loop algorithms proceed by iterating between steps that augment the vectors of locations with known species identities $s_{1},s_{2},\ldots ,s_{K}$ with locations $a_{1}\in u,a_{2}\in u,\ldots ,a_{K}\in u$, update the quadrature weights, and fit point process models as follows:

1. Fit *K* initial point process models using the vectors of observed locations with known species identity $s_{1},s_{2},\ldots ,s_{K}$.
2. Compute the predicted intensities ${\hat{\mu }}_{i}\left(s\right)$ for all $s\in \left\{s\cup u\right\}$ for $i\in \left\{1,\ldots ,K\right\}$ maximizing the likelihood in ((2)).
3. Derive an $\left(M+N\right)\times K$ matrix of membership probabilities **ω**, where

$$\omega =\left[\begin{array}{cccc} \omega_{1}\left(s_{1}\right) & \omega_{2}\left(s_{1}\right) & \ldots & \omega_{K}\left(s_{1}\right)\\ \omega_{1}\left(s_{2}\right) & \omega_{2}\left(s_{2}\right) & \ldots & \omega_{K}\left(s_{2}\right)\\ \vdots & \vdots & \ldots & \vdots \\ \omega_{1}\left(s_{M+N}\right) & \omega_{2}\left(s_{M+N}\right) & \ldots & \omega_{K}\left(s_{M+N}\right) \end{array}\right]$$

The membership probability of location *s* for species *i* is defined as

(3) $$\omega_{i}\left(s\right)=\left\{\begin{array}{cc} 1\left(s\in s_{i}\right) & :s\in s\\ \frac{{\hat{\mu }}_{i}\left(s\right)}{\sum_{i=1}^{K}{\hat{\mu }}_{i}\left(s\right)} & :s\in u \end{array}\right)$$

That is, the membership probabilities for the locations with known species identity are 1 for the correct species and 0 otherwise, and for the locations with unknown species identity, they are proportional to the fitted intensities.

**FIGURE 2:**
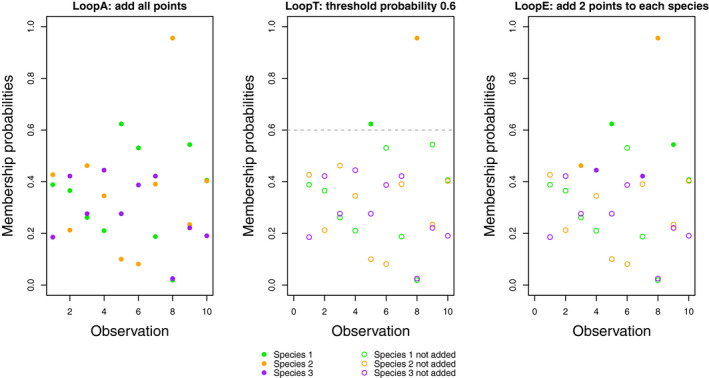
Points added to each species are represented by full circles, the ones that we do not add are open circles. (Left) LoopA function. We add all points with unknown species labels to each species. (Middle) LoopT function. We add all points with membership probabilities higher than the current threshold $\delta_{h}$, set to 0.6 in the middle panel. (Right) LoopE function. We add the points $a_{h}$ with the highest membership probabilities for each species, illustrated for $a_{h}$ = 2 in the right panel

4. Define an augmented vector for species *i* as $y_{i}=s_{i}\cup a_{i}$ for all $i\in \left\{1,\ldots ,K\right\}$, where $a_{i}$ consists of a subset of the vector of observations with unknown species labels **u**. We define $a_{i}$ as follows
• For the **LoopA** method, $a_{i}=u$ (left panel of Figure 2). The A in LoopA reflects the fact that we add all points in **u**.
• For the **LoopT** method, $a_{i}=u_{\left[\omega_{i}\left(s\right)\geq \delta_{h}\right]}$, where $\delta_{h}$ is a minimum membership probability threshold that takes the following values successively at each iteration {$\delta_{\text{max}},\delta_{\text{max}}‐\delta_{\text{step}},\ldots ,\delta_{\text{min}}$ }. That is, the LoopT method augments the locations with known species identity *i* (**s** *_i_*) with the subset of locations with unknown species identity (**u**) that have membership probabilities that are higher than the current threshold $\delta_{h}$ for the species *i* (middle panel of Figure 2). The T in LoopT reflects the fact that we add points with membership probabilities above a certain threshold.
• For the **LoopE** method, $a_{i}=u_{\left[\omega_{i}\left(s\right)\geq \omega_{i,\left(M+N‐a_{h}+1\right)}\right]}$, where $\omega_{i,\left(j\right)}$ represents the *j*th smallest entry of vector $\omega_{i}$, the *i*th column of **ω**, and *a_h_* represents the number of locations to be augmented. That is, the LoopE method augments the locations with known species identity *i* (**s** *_i_*) with the subset of locations with unknown species identity (**u**) with the *a_h_* highest membership probabilities (right panel of Figure 2). The E in LoopE reflects the fact that we add an equal number of point for each species.
5. Update the quadrature weights for each species. First, assign each location in $\left\{y_{1},\ldots ,y_{K},q\right\}$ to a grid cell. Then, compute the vector of quadrature weights $w_{i}$ for all points $t\in \left\{y_{i}\cup q\right\}$ as follows

(4) $$w_{i}\left(t\right)=\frac{c_{1}\times c_{2}\times \omega_{i}\left(t\right)}{1+\sum_{s\in \left\{y_{i}\cup q\right\}}1\left(c\left(s\right)=c\left(t\right)\right)\omega_{i}\left(s\right)}$$

This derivation of the quadrature weights is an extension of standard quadrature weight schemes for point process models (Berman & Turner, 1992), in which the weight for location *s* is equal to the area of the grid cell *c*(*s*) that contains *s* divided by the total number of quadrature and observed locations in *c*(*s*). Here, we define the quadrature weight of the point at location *t* to be the product of the point's membership probability for the given species by the area of the grid cell, divided by the sum of the membership probabilities of the observed locations in the grid cell (both with and without known species identities) plus 1 (for the one quadrature point in the grid cell).

6. Fit point process models using the augmented vector **y** *_i_*, quadrature points **q** and quadrature weights **w** *_i_* for all species $i\in \left\{1,\ldots ,K\right\}$
7. Return to step 2 and stop when we either reach likelihood convergence or we reach a maximum number of iterations that is different depending on the method chosen. Likelihood convergence is determined by:

(5) $$\Delta_{\ell_{h}}=\frac{\sum_{i=1}^{K}\left|\ell_{h}^{i}\left(\beta \right)‐\ell_{h‐1}^{i}\left(\beta \right)\right|}{\sum_{i=1}^{K}\ell_{h‐1}^{i}\left(\beta \right)}<\varepsilon$$

for some choice of ε, where $\ell_{h}^{i}\left(\beta \right)$ is the fitted log‐likelihood for the *i*th species at the *h*th iteration.

The maximum number of iterations varies for the different methods, as follows:

- For the **LoopA** method, the maximum number of iterations is set by the user. We set the default number of iterations to be 50.
- For the **LoopT** method, the maximum number of iterations is determined by:

(6) $$\frac{\delta_{\max}‐\delta_{\min}}{\delta_{\text{step}}}+1$$

- For the **LoopE** method, the maximum number of iterations is $\left\lfloor \frac{N}{K}\right\rfloor ‐a_{1}$, where $\left\lfloor c\right\rfloor$ rounds the number *c* down to the nearest integer, and *a*_1_ is the first value of *a_h_* chosen by the user. In the case of decimal numbers, only the floor is considered as we cannot add more points than available per species.

### 2.4 Mixture of PPM method

Mixture methods can be fitted by maximizing a log‐likelihood function and reclassifying the locations with uncertain identity using an EM algorithm framework. We developed the tool such that both soft and hard classification methodology are available. Various initialization schemes can be used, and we have chosen to use four different schemes, described below:

1. Initialize the membership probabilities **ω** for each location *s* for each species *i* in one of the following ways
• For the **knn method**, we calculate the distance of the unknown labeled location *u* to all the point locations **s**. For each *u*, we consider the *k* closest neighbors in **s** regardless of species. Then, we calculate the membership probability of location *s* for species *i* using:

(7) $$\omega_{i}\left(s\right)=\left\{\begin{array}{cc} 1\left(s\in s_{i}\right) & :s\in s\\ \frac{z_{i}\left(s\right)}{\sum_{i=1}^{K}z_{i}\left(s\right)} & :s\in u \end{array}\right)$$

where

(8) $$z_{i}\left(s\right)=\sum_{k}\min_{k}\frac{1}{d_{i,k}\left(s\right)}$$

where the $d_{i,k}\left(s\right)$ are the *k*th distances for the species *i* at the location *s*.

- For the **kmeans method**, we define $\omega_{i}\left(s\right)$ as in ((7)) but define $z_{i}\left(s\right)$ as

(9) $$z_{i}\left(s\right)=\frac{\text{min}\left(d_{i}^{C}\left(s\right)\right)}{d_{i}^{C}\left(s\right)}$$

where $d_{i}^{C}\left(s\right)$ is the distance to the *i*th centroid of the *i*th cluster. The kmeans initialization is performed by the kmeans function in R, where we repeat the initialization multiple times as defined by the parameter nstart in R.

- For the **random method**, we define $\omega_{i}\left(s\right)$ as in ((7)) and $z_{i}\left(s\right)$ is drawn randomly from a uniform distribution:

(10) $$z_{i}\left(s\right):U\left[0,1\right]$$

The random method is used as an uninformative approach for comparison to other methods.

- For the **CoinF**, we set the initial membership probabilities as follows:

(11) $$\omega_{i}\left(s\right)=\left\{\begin{array}{cc} 1 & :s\in y_{i}\\ 0 & \;:\text{otherwise} \end{array}\right)$$

where we define the augmented vector **y** *_i_* similarly to Step 4 of the Loop algorithm in Section 2.3 and **a** *_i_* is defined as the vector of observations with unknown species labels randomly assigned to one of the species.

Regardless of the initialization method, the sum of membership probabilities across the species is equal to 1 for all points.

2. For soft classification: Create a list of point patterns, one for each species, each containing the locations with known identity **s** *_i_* as well as the locations of the observations with unknown identity **u**. For each point pattern, we define the quadrature weights as in ((4)), using the membership probabilities $\omega_{i}$ defined in Step 1.

For hard classification: Assign the locations in **u** to belong to one of the *K* species based on the membership probabilities **ω**. The classification is based on the highest membership probability.

3. Fit a point process model for each pattern defined in Step 2.
4. E step: Compute the predicted intensities ${\hat{\mu }}_{i}\left(s\right)$ for each species.
5. Calculate the predicted intensity of the mixture of *K* densities using:

(12) $$\nu \left(s\right)=\sum_{i=1}^{K}\nu_{i}\left(s\right)=\sum_{i=1}^{K}\pi_{i}\times {\hat{\mu }}_{i}\left(s\right)$$

Here, ${\hat{\mu }}_{i}\left(s\right)$ is the intensity at location *s* for the *i*th species and $\pi_{i}$ is the mixing proportion or weight of the *i*th species and is given by:

(13) $$\pi_{i}=\frac{\sum_{s\;\in \;y_{i}}\omega_{i}\left(s\right)}{\sum_{i=1}^{K}\sum_{s\;\in \;y_{i}}\omega_{i}\left(s\right)}$$

where $\omega_{i}\left(s\right)$ represents the membership probability of the *i*th species at the location *s*. The resulting $\nu_{i}\left(s\right)$ is thus the mixture intensity of the *i*th species.

6. Update the membership probabilities for the locations with unknown species identity **u** using

(14) $$\omega_{i}\left(s\right)=\left\{\begin{array}{cc} 1\left(s\in s_{i}\right) & :s\in s\\ \frac{\nu_{i}\left(s\right)}{\sum_{i=1}^{k}\nu_{i}\left(s\right)} & :s\in u \end{array}\right)$$

7. For soft classification, M step: Update the quadrature weights for all locations in **s** and **u** as in Step 2. If any location with an unknown label u∈u has a membership probability of $\omega_{i}\left(u\right)$ = 0 for species *i*, that location is removed from the point pattern of species *i* before proceeding to the next step for the current iteration.

For hard classification, M step: Assign the locations in **u** to belong to one of the *K* species. The classification for each point *s* corresponds to the highest membership probability $\omega_{i}\left(s\right)$ for $i\in \left\{1,\ldots ,K\right\}$.

We compute each species' proportion of the whole by summing the membership probabilities for each species across both **s** and **u**.

8. For soft classification, fit an updated PPM using the updated quadrature weights and membership probabilities

For hard classification, compute a marked PPM based on the updated classifications.

9. Calculate the model log‐likelihood using

(15) $$\ell \left(\beta \right)=\sum_{s\in s\cup u}f\left(s,\beta \right)=\sum_{s\in s\cup u}\ln\sum_{i=1}^{K}\pi_{i}\times \nu_{i}\left(s,\beta_{i}\right)$$

where $f\left(s,\beta \right)$ is the mixture density function defined at locations s∈s∪u and parameterized by **β**.

10. Repeat steps 4–9 until we achieve likelihood convergence, defined as follows

(16) $$\frac{\left|\ell_{h}\left(\beta \right)‐\ell_{h‐1}\left(\beta \right)\right|}{\left|\ell_{h‐1}\left(\beta \right)\right|}<\varepsilon$$

where $\ell_{h}\left(\beta \right)$ is the log‐likelihood at the *h*th iteration and ε is a prespecified tolerance level.

When the model has converged, we use hard classification for the locations **u** with unknown species identity when evaluating model performance.

## 3 SIMULATION FRAMEWORK AND APPLICATION

### 3.1 Simulation data

To compare the performance of the different algorithms, we simulated patterns **t**_1_, **t**_2_, and **t**_3_ of individuals for three species based on “true” distributions defined by four different predictors. Because performance could vary based on sample size, the correlations $\rho_{i‐j}$ among the species distributions, and the proportion of observations with unknown labels, we simulated point patterns in which relative abundance patterns and correlation among distributions vary. In summary, we tested the following cases:

- test 1: $m_{1}$ = 80, $m_{2}$ = 60, $m_{3}$ = 40; $\rho_{1‐2}$ = 0.09, $\rho_{1‐3}$ = −0.42, $\rho_{2‐3}$ = 0.20;
- test 2: $m_{1}$ = 60, $m_{2}$ = 60, $m_{3}$ = 60; $\rho_{1‐2}$ = 0.09, $\rho_{1‐3}$ = −0.42, $\rho_{2‐3}$ = 0.20;
- test 3: $m_{1}$ = 80, $m_{2}$ = 60, $m_{3}$ = 40; $\rho_{1‐2}$ = 0.85, $\rho_{1‐3}$ = −0.09, $\rho_{2‐3}$ = 0.20;
- test 4: $m_{1}$ = 60, $m_{2}$ = 60, $m_{3}$ = 60; $\rho_{1‐2}$ = 0.85, $\rho_{1‐3}$ = −0.09, $\rho_{2‐3}$ = 0.20.

We chose low values for abundances as they would be small enough such that potential value of adding points with unknown species identities could be investigated. We chose these cutoffs for correlation to create clearly distinguishable contexts. We note that species are independent, and we do not investigate interactions between the species.

We then created locations with unknown labels **u** by hiding uniformly at random a certain proportion of the total observations (20%, 50% and 80%). The locations in $t_{1}$, $t_{2}$, and $t_{3}$ that retained their true species identities therefore became the simulated point patterns $s_{1}$, $s_{2}$, and $s_{3}$ with known species identities. The hidden points form **u**.

Simulations were conducted using the version 4.0.2 of R (R Development Core Team, 2017). We implemented 1,000 simulations of each of the 4 sets of test patterns previously described using a high performance computing cluster from the University of Newcastle, on 512 GB nodes powered by 3.0 GHz Intel Xeon Gold (E5‐6154) processors. We display the Hard classification implementation hereafter because it showed the best performances. We tested the effects of different parameters on method performance for the following methods:

- knn: the value of k neighbors,
- kmeans: the number of random initializations nstart,
- LoopT: the maximum threshold $\delta_{\text{max}}$, minimum threshold $\delta_{\text{min}}$ and the step size $\delta_{\text{step}}$,
- LoopE: initial number of points added to the point pattern $a_{1}$.

We show how these parameters affect the performance in Appendix A.

### 3.2 Suite of evaluation tools

We considered various measures of performance for comparing the distributions. For classification methods, misclassification/accuracy analysis is a common measure of performance (Wendel et al., 2015). We chose the highest membership probability for each observation to determine the labeling of hidden points and compared it with its true label when computing the accuracy:

(17) $$\text{Accuracy}=\frac{\text{Number of correct labels}}{N}$$

where N is the number of observations with uncertain species identities.

We also compared the final membership probabilities of the reclassified point labels of each point to 1 (the true weight) with a residual sum of squares (RSS).

(18) $$\text{RSS}=\sum_{i=1}^{K}\sum_{s\in u\cap t_{i}}{\left(\omega_{i}\left(s\right)‐1\right)}^{2}$$

where $\omega_{i}\left(s\right)$ is the final membership probability for location s for the reclassified point of species i computed using the methods outlined in Sections 2.3 and 2.4. Considering residual sum of squares (RSS) alone does not provide a reliable comparison because the number of unknown observations can vary, so we considered meanRSS instead to standardize the measure for all fitted models:

(19) $$\text{meanRSS}=\frac{\text{RSS}}{N}$$

We also obtained these performance measures for models fitted using only the locations with known species identity, hereafter referred to as the “individual PPM” method. In this way, we have a baseline with which to judge whether the mixture and Loop methods outperform the standard approach of discarding points with unknown species identity.

We also computed performance measures based on predicted intensities. We compared the true distribution from which we generated the points to the predicted distributions of the various models we fitted. We used a sum of correlations between the true and predicted distributions across all species (hereafter referred to as “sumcor”) to assess how well the predicted distributions align with the true distributions. We can use various correlation measures such as Pearson's correlation coefficient, Kendall's τ, or Spearman's ρ when computing sumcor.

Another global measure of predictive performance of the intensity estimates is the Integrated Mean Square Error (IMSE) (Es, 1997; Swanepoel, 1988). The function is defined as:

(20) $$\text{IMSE}=E\left(\int_{‐\infty }^{+\infty }{\left({\hat{f}}_{n}\left(x\right)‐f\left(x\right)\right)}^{2}dx\right)$$

where ${\hat{f}}_{n}\left(x\right)$ is an estimator of the density function $f\left(x\right)$. Because the scale of the IMSE depends on the magnitude of the true intensity, we rescaled both true and predicted intensities to have a common mean to make for an equitable comparison. We computed the IMSE using the values of the true and predicted intensities at the quadrature points **q**, and sum across the three species (sumIMSE):

(21) $$\text{sumIMSE}=\sum_{i=1}^{K}\sum_{q=1}^{Q}{\left({\hat{\mu }}_{i}^{{\bar{t}}_{i}}\left(s_{M+N+q}\right)‐\mu_{i}\left(s_{M+N+q}\right)\right)}^{2}$$

where ${\hat{\mu }}_{i}^{{\bar{t}}_{i}}\left(s\right)$ is the predicted intensity of species i at location s rescaled to have mean ${\bar{t}}_{i}$. We also displayed the standard error of the point predictions as a measure of uncertainty. We weight the standard error measure by the number of points for an equitable comparison across different percentages of hidden observations.

### 3.3 Application to eastern Australian frogs

Our study case dataset uses presence‐only records from the online database of the Atlas of Living Australia (ALA, 2018). On this platform, any person that sees a frog in the wild can report the coordinates and other relevant information. We focused the analysis on the three northern species of *Mixophyes* genus that have been recently separated in Mahony et al. (2006). We cleaned our dataset by including only observations of adult specimens with date information and through verification by a specialist of these species, M. Mahony. The observations with known species labels were those for which we have associated genetic information as well as any observations reported after the taxonomic split in 2006. The rest of the observations were considered as having unknown species labels. We also included data from Oza et al., (2012) as part of the known labeled points. Altogether, we count 181 out of the 444 observations with unknown labels (approximately 40.8%).

We extracted relevant covariates for these species on a 5 km × 5 km grid from different sources as presented here (Table 1).

**TABLE 1:** Description and origin of the different covariates used in the analysis of the *Mixophyes* dataset

Name	Description	Source
Bio05	Max Temperature of Warmest Month	BBCVL
Bio06	Min Temperature of Coldest Month	BBCVL
Bio11	Mean Temperature of Coldest Quarter	BBCVL
Bio13	Precipitation of Wettest Month	BBCVL
Bio18	Precipitation of Warmest Quarter	BBCVL
Altitude	Altitude	BBCVL
Dist road	Distance to the nearest roads	UC Davis Biogeo group
Dist stream	Distance to the nearest hydrological features	Bureau of Meteorology (2014)

We fitted models using the methods that performed best in the simulation study and compared them with the individual PPM method for which no points with unknown labels were used.

## 4 RESULTS

Here, we present the model performances on the simulated data parameters (abundance, correlation, and percentage of points with hidden species labels). We explore the role of different parameters within the various mixture and loop methods in Appendix A. The individual PPM results will be used as a point of comparison with the other methods as the individual PPM method does not include any of the points with unknown labels. We choose to use Pearson's correlation coefficient when computing sumcor. We conclude the section by comparing maps and membership probabilities of the *Mixophyes* species.

### 4.1 Testing species distributions

In this section, we compare the results of varying abundance, the correlation between species distributions, and the percentage of hidden observations on the performance measures and membership weights for classification as presented in Section 3. We only present the best‐performing methods in this section: knn mixture, LoopA, LoopT, LoopE, and the individual PPM and coinF method for reference.

#### 4.1.1 Relabeling performance measures

**FIGURE 3:**
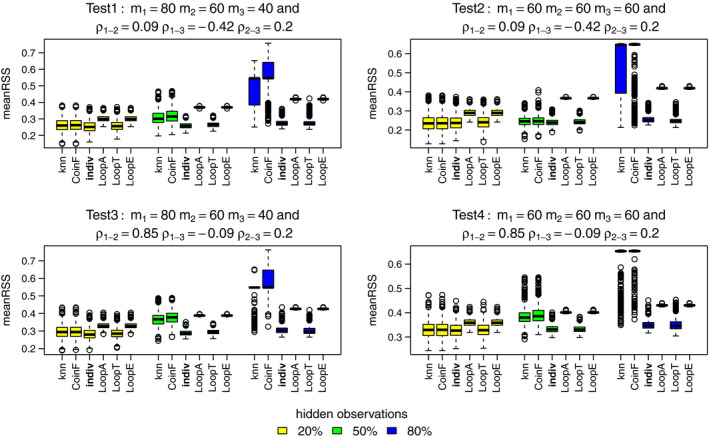
MeanRSS for the best methods: knn, coinF, individual PPM (reference), LoopA, LoopT, and LoopE. Each color boxplot represents a different percentage of hidden observations: in yellow are the performances for 20% of hidden observations, in green for 50% and in blue for 80%. For each method, we fitted models to the three simulated point patterns using four simulated predictors. A low meanRSS value indicates a high performance

In terms of relabeling, only LoopT consistently performs as well or better than the individual PPM method across all simulation designs and percentage of hidden observations, as shown in Figure 3. The mixture methods are more competitive than the LoopA and LoopE methods at 20% and 50% of hidden observations but still do not perform as well as the individual PPM or LoopT methods.

**FIGURE 4:**
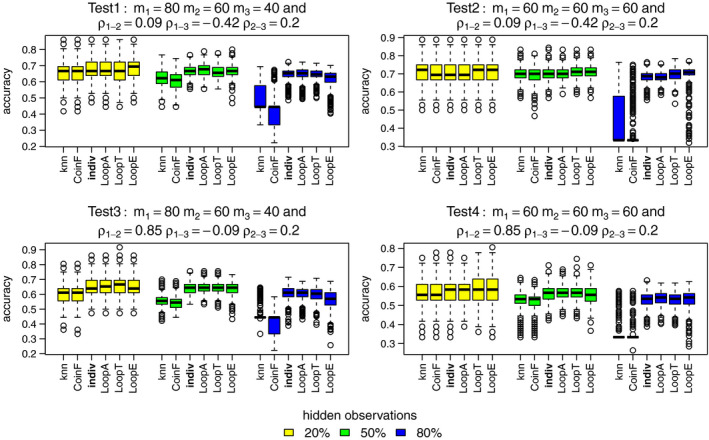
Accuracy for the best methods: knn, coinF, individual PPM (reference), LoopA, LoopT, and LoopE. Each color boxplot represents a different percentage of hidden observations: in yellow are the performances for 20% of hidden observations, in green for 50% and in blue for 80%. For each method, we fitted models to the three simulated point patterns using four simulated predictors. A high accuracy value indicates a high performance

Comparing accuracy, all three Loop methods perform comparably to the individual PPM method. The knn and coinF methods are equally competitive at 20% of hidden observations but their performances get worse than the other methods for 50% and 80% percentages in Figure 4.

#### 4.1.2 Predicted intensity performance measures

**FIGURE 5:**
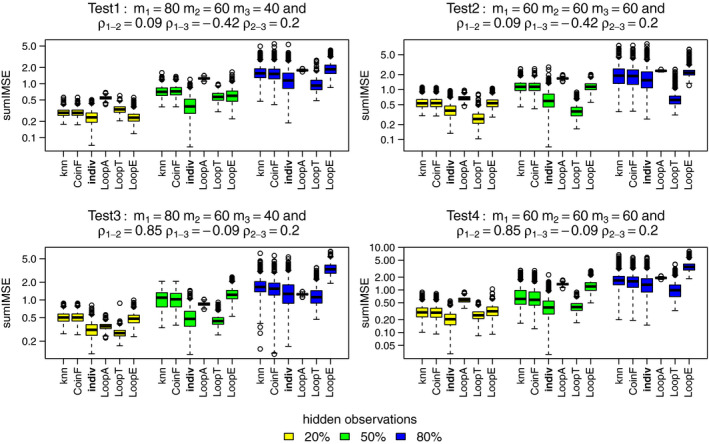
SumIMSE (logarithmic scale) for the best methods: knn, coinF, individual PPM (reference), LoopA, LoopT, and LoopE. Each color boxplot represents a different percentage of hidden observations: in yellow are the performances for 20% of hidden observations, in green for 50% and in blue for 80%. For each method, we fitted models to the three simulated point patterns using four simulated predictors. A low sumIMSE value indicates a high performance

Now, we consider performance based on predicted intensity. The LoopT method performs as well or better than the individual PPM method according to sumIMSE as shown in Figure 5. The LoopA, LoopE, knn, and CoinF methods are mostly never competitive with the other methods at high percentage of hidden observations.

**FIGURE 6:**
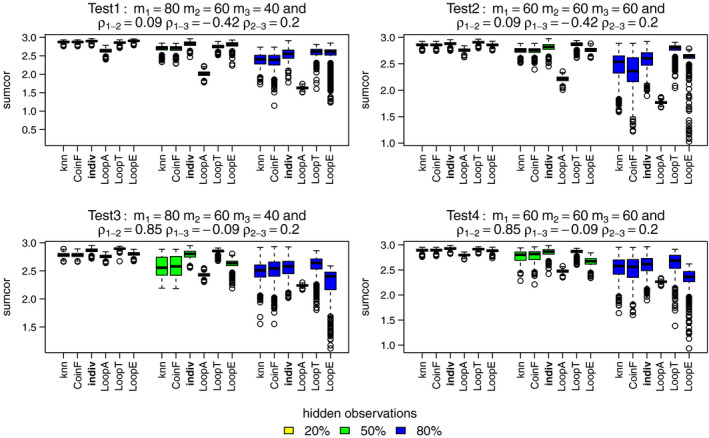
Sumcor for the best methods: knn, coinF, individual PPM (reference), LoopA, LoopT, and LoopE. Each color boxplot represents a different percentage of hidden observations: in yellow are the performances for 20% of hidden observations, in green for 50% and in blue for 80%. For each method, we fitted models to the three simulated point patterns using four simulated predictors. A high sumcor value indicates a high performance

The relative performance is different when using sumcor as the performance measure as shown in Figure 6. It looks like LoopT is consistently best, and the individual PPM method and LoopE methods are broadly comparable for nonhighly correlated distributions. The knn and coinF methods perform almost equally to the individual PPM method when a relatively low percentage of observations have hidden labels and when distributions are highly correlated.

Comparisons of the estimated standard errors appear in Appendix A. Standard errors for the predicted intensities increase, as expected, when the number of observations used in the models decreases, as shown in Figures A5 and A6. This is evident from the higher standard errors for higher percentages of observations with hidden labels as well as for the individual PPM method, which does not add any points.

#### 4.1.3 Final membership probabilities and classification

**FIGURE 7:**
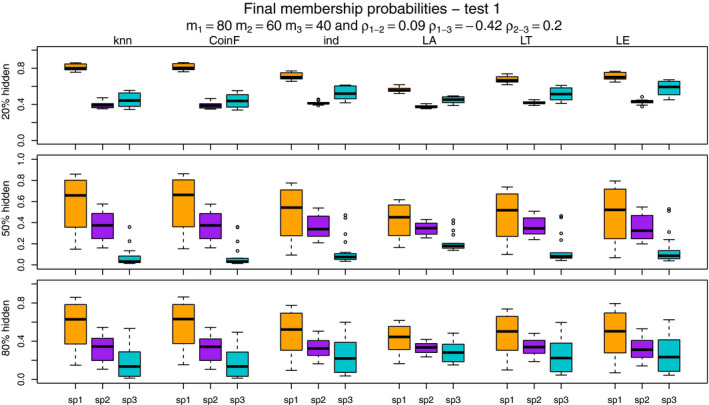
The boxplots display the estimated membership probabilities of the correct species for points with hidden labels in test 1. Each color boxplot represents a different species. Each row corresponds to the different percentage of hidden observations tested: 20%, 50%, and 80%. Test 1 is based on simulated point patterns with abundances of $m_{1}$ = 80, $m_{2}$ = 60, $m_{3}$ = 40; and correlations between the species distributions of $\rho_{1‐2}$ = 0.09, $\rho_{1‐3}$ = −0.42, $\rho_{2‐3}$ = 0.20

**FIGURE 8:**
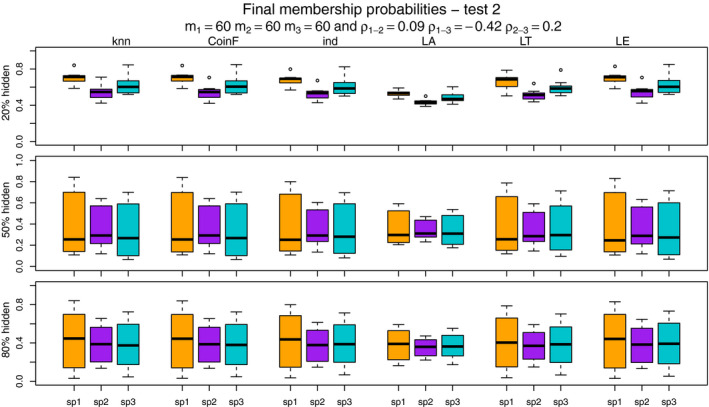
The boxplots display the estimated membership probabilities of the correct species for points with hidden labels in test 2. Each color boxplot represents a different species. Each row corresponds to the different percentage of hidden observations tested: 20%, 50%, and 80%. Test 2 is based on simulated point patterns with abundances of $m_{1}$ = 60, $m_{2}$ = 60, $m_{3}$ = 60; and correlations between the species distributions of $\rho_{1‐2}$ = 0.09, $\rho_{1‐3}$ = −0.42, $\rho_{2‐3}$ = 0.20

**FIGURE 9:**
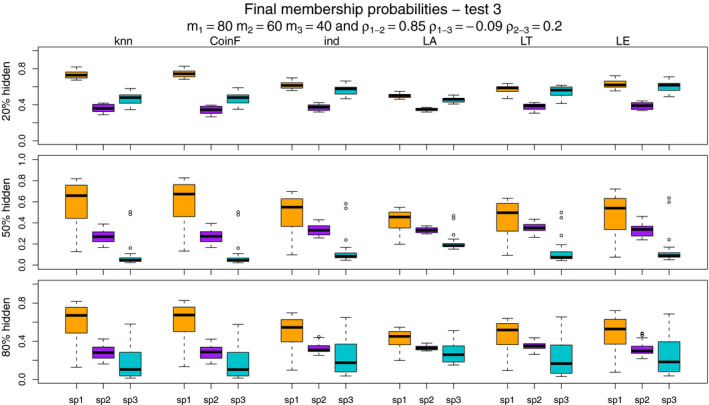
The boxplots display the estimated membership probabilities of the correct species for points with hidden labels in test 3. Each color boxplot represents a different species. Each row corresponds to the different percentage of hidden observations tested: 20%, 50%, and 80%. Test 3 is based on simulated point patterns with abundances of $m_{1}$ = 80, $m_{2}$ = 60, $m_{3}$ = 40; and correlations between the species distributions of $\rho_{1‐2}$ = 0.85, $\rho_{1‐3}$ = −0.09, $\rho_{2‐3}$ = 0.20

**FIGURE 10:**
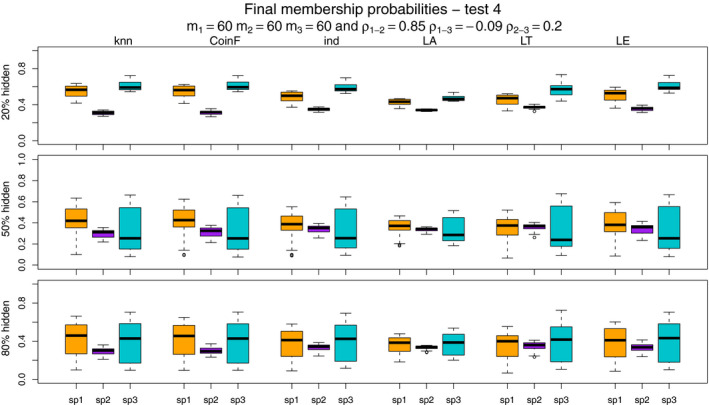
The boxplots display the estimated membership probabilities of the correct species for points with hidden labels in test 4. Each color boxplot represents a different species. Each row corresponds to the different percentage of hidden observations tested: 20%, 50%, and 80%. Test 4 is based on simulated point patterns with abundances of $m_{1}$ = 60, $m_{2}$ = 60, $m_{3}$ = 60; and correlations between the species distributions of $\rho_{1‐2}$ = 0.85, $\rho_{1‐3}$ = −0.09, $\rho_{2‐3}$ = 0.20

Figures 7, 8, 9, 10 show the final membership probabilities of the locations with hidden species identity corresponding to each species. The higher the membership probability is to 1, the better the classification performance. It appears that the high correlation among the species distributions as in tests 3 and 4 results in lower classification performance. When there are differences in abundance (test 1 and test 3), the mixture methods seem to show superior performance for the most abundant species and worse performance for the least abundant species.

### 4.2 The *Mixophyes* case

#### 4.2.1 Prediction of *Myxophies*' species distribution

**FIGURE 11:**
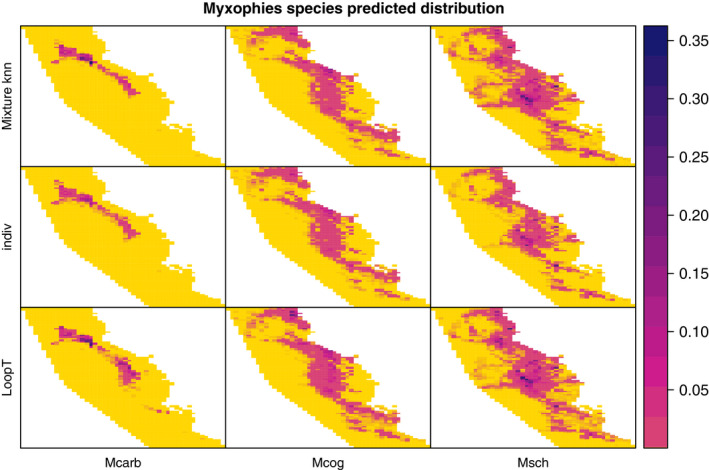
Distribution of the *Mixophyes* species predicted intensities for the mixture knn initialization method, the individual PPM method without the reclassified points and the LoopT method. Mcarb stands for *Mixophyes carbinensis*, Mcog stands for *Mixophyes coggeri,* and Msch stands for *Mixophyes schevilli*

In this section, we fit the best‐performing method within each category (knn among the mixture methods and LoopT among the Loop methods) to analyze the distribution of the *Mixophyes* species and compare the predictions to the individual PPM approach in which no unlabeled observations are included in the model. The resulting fitted intensity maps are shown in Figure 11. Both the knn mixture method and the LoopT method add small areas of distribution for *Mixophyes schevilli*. The maps from the LoopT method show increased areas of relatively high intensity in the south for *Mixophyes carbinensis* and *Mixophyes coggeri*.

#### 4.2.2 Classification of *Myxophies* observations

**FIGURE 12:**
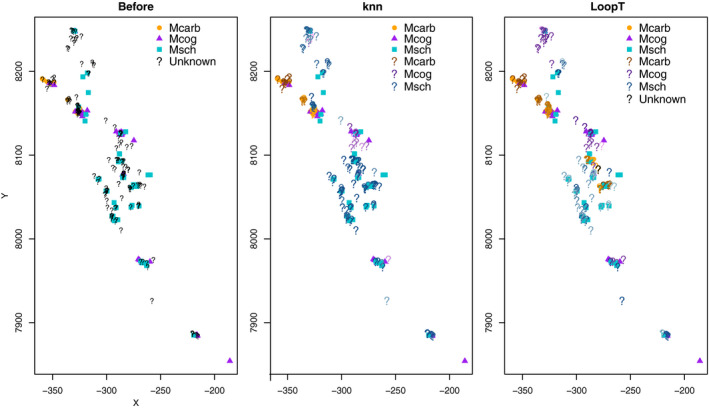
Observed locations for the *Mixophyes* data set. On the left, the points with unknown species labels have not been classified. The remaining maps show the final classification for the knn mixture (middle) and LoopT (right) methods. The orange dots, purple triangles, and blue squares represent labeled points, while the question marks represent the points with unknown labels. The color of the question marks indicates their classification, with black representing an unclassified point, and the intensity of colored question marks representing the final membership probabilities, with bolder colors representing higher probabilities

Differences in the predicted distributions are also shown by the classification of the locations with uncertain identities in Figure 12. While there is broad agreement in the south for the knn mixture method and the LoopT method, the LoopT method classifies more records as *M. coggeri* in the north and *M. carbinensis* in the central part, while the knn mixture method classifies more records as *M. schevilli* in the north and central parts. This may reflect the fact that the mixture methods tend to have high classification for the most abundant species, and *M. schevilli* had the highest number of verified records among the three species.

**FIGURE 13:**
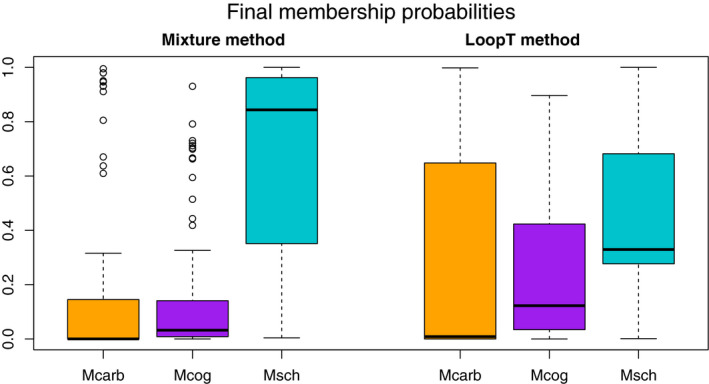
Final membership probability per species for the knn mixture method and the LoopT method. Each color represents a different species: Mcarb; *M. carbinensis*, Mcog; *M. coggeri* and Msch; *M. schevilli*

The colors of the question marks in Figure 12 are based on the final membership probabilities, with higher membership probabilities leading to bolder colors. This Figure indicates that the mixture knn method tends to result in lower membership probabilities than the LoopT method except for the most abundant species *M. schevilli*, which is also supported by Figure 13, in which the final membership probabilities for the LoopT method tend to be more variable, with the third quartile markedly higher for each species. The final membership probabilities appear more balanced for the LoopT method, whereas the knn mixture method tends to favor the most abundant species, *M. schevilli*.

## 5 DISCUSSION

In this article, we present a new modeling framework implemented in R that aims to incorporate the observed locations with unknown species identities to improve species distributions. These tools accommodate two ways of reclassifying information using mixture modeling and a machine learning framework with 7 different implementation methods overall. We tested our algorithms in different contexts where we vary the abundances of our species (equal across the species or different), the correlation between them (two distributions are highly correlated or all have low correlation), and the proportion of unknown species identities (20%, 50%, and 80%). We compared our methods with the individual PPM method which ignores locations with unknown species identities to see whether the proposed algorithms allow us to fit distributions that are closer to the initial processes.

The novelty of these methods makes it difficult to compare to other existing tools that do not combine point pattern processes, mixture models, or semisupervised learning methods. Most mixture models also use the EM algorithm but are implemented for Gaussian mixture distributions only (Benaglia et al., 2009; Di Zio et al., 2007; Quost & Denoeux, 2016; Scrucca et al., 2016). A few implementations use both mixture and semisupervised learning but do not use presence‐only data or point pattern processes (Figueirido & Jain, 2002; Frame & Jammalamadaka, 2007; Melnykov & Maitra, 2010; Woillez et al., 2012). Flexible R packages such as Flexmix (Leisch, 2004), mixtools (Benaglia et al., 2009) and MixAll (Iovleff, 2018) are not suitable to our design. The work of Taddy and Kottas (2012) is noteworthy in that it models a mixture of marked point processes in a Bayesian framework, but it does not allow for semisupervised learning and therefore cannot accommodate settings such as ours in which some points have unknown species labels. However, as the goal is to investigate whether there is any benefit from adding points with unknown species labels when fitting models, comparison to the individual PPM method which does not add any unlabeled points allows us to compare the proposed methods to a natural baseline.

In our simulations, we have considered a relatively general case of point patterns and we only varied species abundance and correlation among distributions in addition to the proportion of observations with hidden information. The results show that some methods benefit from adding points with unknown species labels, leading to improved performances. We noticed a discrepancy in performances that is more significant when we increase the proportion of observations with hidden labels. While at 20% of hidden observations, all methods performed fairly similarly, at 50% and 80% of hidden observations the Loop methods performed the best. In particular, the LoopT method showed consistently good performances across all measures studied. For this method, only the points with the highest membership probabilities are added. We explore the roles of the $\delta_{h}$ parameters in Appendix A. We set the maximum and minimum thresholds at $\delta_{\text{max}}=0.5$ and $\delta_{\text{min}}=0.1$ and a step size of $\delta_{\text{step}}=0.1$ as it appears to be the best combination. LoopE showed competitive results to LoopT or the individual PPM method looking at predictive performances in the case of not highly correlated distributions, but may not be able to distinguish highly correlated distributions. For this method, we add the $a_{h}$ points with highest membership probabilities, with the number of points $a_{h}$ increasing at each iteration. We explore the role of this parameter in Appendix A. For the LoopA method, we add all unknown points to the known points; thus, the reclassification and density distribution can be biased by the unknown points not belonging to a certain species. The LoopA method displayed similar results as the mixture methods for predictive performances (sumIMSE) but outperformed these methods in relabeling.

**FIGURE A1:**
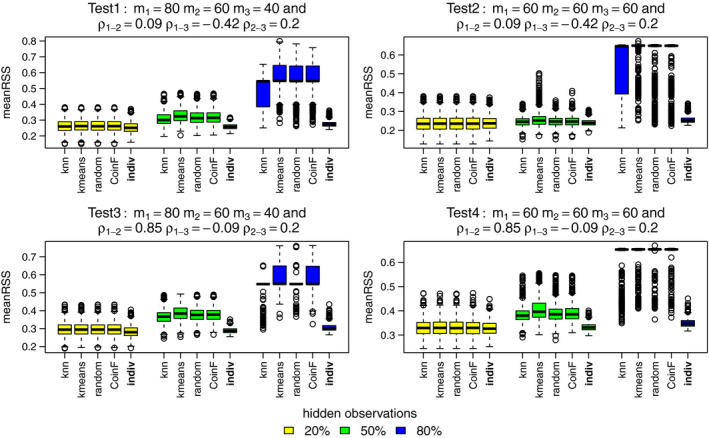
MeanRSS for the methods: knn, kmeans, random, coinF, and individual PPM (reference). Each color boxplot represents a different percentage of hidden observations: in yellow are the performances for 20% of hidden observations, in green for 50%, and in blue for 80%

**FIGURE A2:**
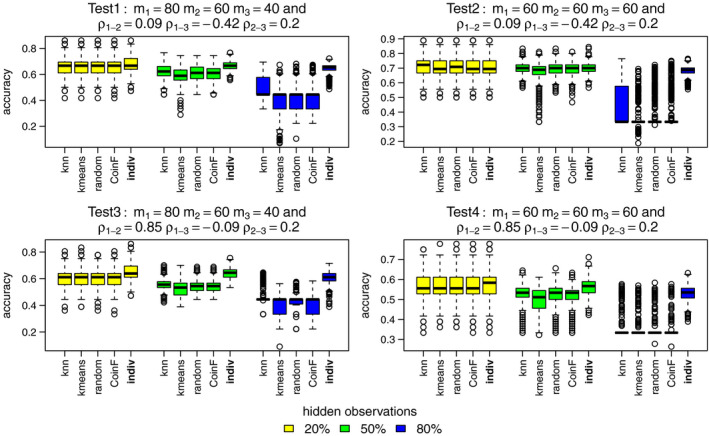
Accuracy for the methods: knn, kmeans, random, coinF, and individual PPM (reference). Each color boxplot represents a different percentage of hidden observations: in yellow are the performances for 20% of hidden observations, in green for 50% and in blue for 80%

**FIGURE A3:**
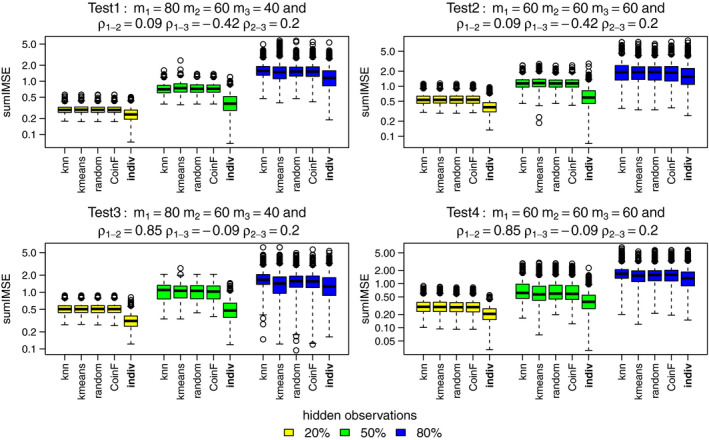
SumIMSE (logarithmic scale) for the methods: knn, kmeans, random, coinF, and individual PPM (reference). Each color boxplot represents a different percentage of hidden observations: in yellow are the performances for 20% of hidden observations, in green for 50%, and in blue for 80%

**FIGURE A4:**
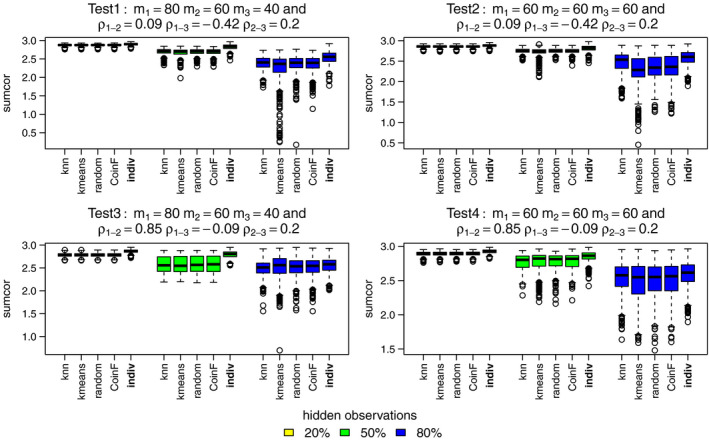
Sumcor for the methods: knn, kmeans, random, coinF, and individual PPM (reference). Each color boxplot represents a different percentage of hidden observations: in yellow are the performances for 20% of hidden observations, in green for 50%, and in blue for 80%

**FIGURE A5:**
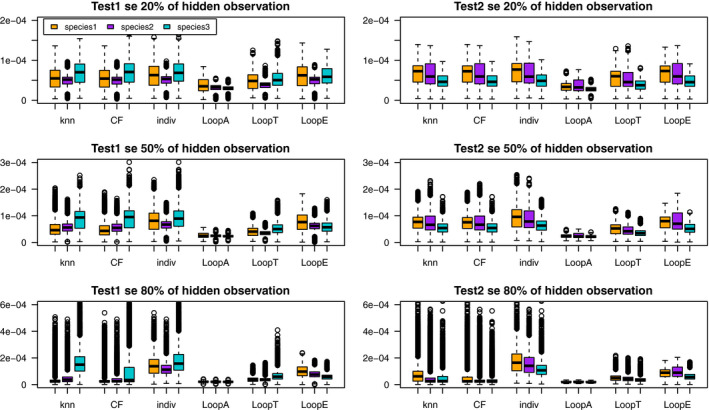
Standard error for the best methods: knn, coinF, individual PPM (reference), LoopA, LoopT, and LoopE. Each color boxplot represents a different species: in orange species 1, in purple species 2, and in turquoise species 3. The tests use the following parameters: test 1: $m_{1}$ = 80, $m_{2}$ = 60, $m_{3}$ = 40; $\rho_{1‐2}$ = 0.09, $\rho_{1‐3}$ = −0.42, $\rho_{2‐3}$ = 0.20; test 2: $m_{1}$ = 60, $m_{2}$ = 60, $m_{3}$ = 60; $\rho_{1‐2}$ = 0.09, $\rho_{1‐3}$ = −0.42, $\rho_{2‐3}$ = 0.20

**FIGURE A6:**
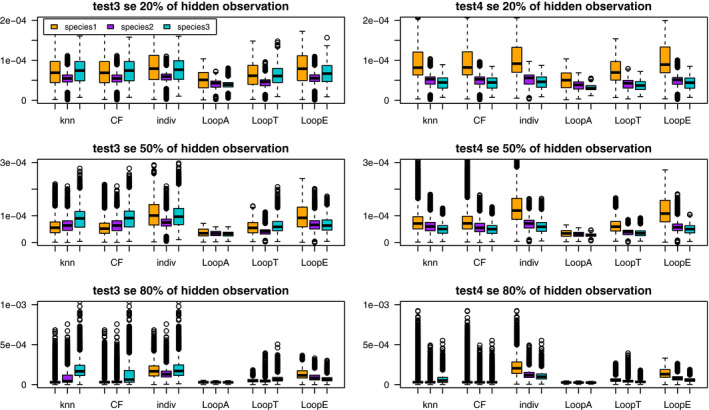
Standard error for the best methods: knn, individual PPM (reference), LoopA, LoopT, and LoopE. Each color boxplot represents a different species: in orange species 1, in purple species 2, and in turquoise species 3. The tests use the following parameters: test 3: $m_{1}$ = 80, $m_{2}$ = 60, $m_{3}$ = 40; $\rho_{1‐2}$ = 0.85, $\rho_{1‐3}$ = −0.09, $\rho_{2‐3}$ = 0.20; test 4: $m_{1}$ = 60, $m_{2}$ = 60, $m_{3}$ = 60; $\rho_{1‐2}$ = 0.85, $\rho_{1‐3}$ = −0.09, $\rho_{2‐3}$ = 0.20

The methods using the mixture algorithm tend to perform worse than the Loop methods and the individual PPM method for moderate and high percentages of hidden observations (50% and 80%). However, mixture methods performed relatively better for highly correlated distributions in their predictive performances, which relate to the mixture methods' ability to distinguish multiple distributions inside one distribution. We note that the mixture methods displayed high membership probabilities for the most abundant species. Indeed, the method makes use of mixing proportions, which give further emphasis to the most dominant species. Hence, they tend to favor the most abundant species while not classifying well the other species with lower abundances. The methods (kmeans, random) not presented previously in the results are presented in Appendix A (see Figures A1, A2, A3, A4). All methods showed relatively similar performance to each other across all measures.

Contrary to what we found, previous studies using the EM algorithm for classification and clustering data show that such algorithms are highly dependent on the initialization method (Figueirido & Jain, 2002; Melnykov & Maitra, 2010; O'Hagan et al., 2012). Studies link the performance of the knn method to the metric chosen to calculate the nearest neighbor distances and the value of the number k of nearest neighbors (Guo et al., 2003; Weinberger & Saul, 2009; Wu et al., 2008). Even the established kmeans method shows drawbacks as its performance depends on overlapping densities and whether the distributions are roughly circular. The choice of the centroid is also not consistent and chosen at random for the first calculation (Wu et al., 2008; Yoo et al., 2012). The coinF method, which randomly assigns species labels, is in line with the other mixture methods and never reaches the performance of the Loop methods. Consequently, we have shown that the loop methods outperform not only the individual PPM method but also a method that randomly assigns species labels. A future research area could look into the different metrics to evaluate nearest neighbors (knn) or the centroid choice (kmeans).

We also tested the best‐performing method LoopT and the knn method on the *Mixophyes* dataset. As mentioned in the results, the knn method will favor the most abundant species of the dataset and in our context assigned more points with unknown species labels to *M. schevilli*, while the LoopT method produced more balanced species assignments. The value of the proposed Mixture and Loop methods is to make use of observations with uncertain species identities, and our results suggest that the LoopT method provides the best combination of accuracy in prediction and classification.

There are more factors to consider for real ecological datasets that can influence how a model will perform. First, the abundances tested in the simulation are quite low (40 points at the lowest) and some methods can show convergence issues in this context. While we use the spatstat package (Baddeley et al., 2015) to fit PPMs, we could make use of similar functions in the ppmlasso package (Renner & Warton, 2013) which integrate regularization methods like the lasso penalty that can boost performances with low sample sizes. In our model, we included all covariates used to generate the true point patterns; however, for real datasets we may not have access to the best covariates or know which ones precisely determine the species distributions. Applying a lasso penalty to help in variable selection may therefore provide a natural way forward in this context. Finally, a key reality when dealing with presence‐only data is the observer bias, for which sampling effort varies throughout the study region. Some models apply a correction for observer bias in the prediction (Hefley et al., 2013; Lahoz‐Monfort et al., 2014; Warton et al., 2013), and our proposed methods could be extended to accommodate these approaches of accounting for observer bias.

## 6 CONCLUSION

The new algorithms presented in this article aim to reclassify observations that have uncertain or unknown labels in order to better predict point pattern distributions. We showed that machine learning‐based models performed better in a general context than mixture ones no matter the initialization method and also better than the individual PPM method that does not include the points with unknown labels. Our simulations showed encouraging results in this context with good performances in some cases. They can be adapted to account for other considerations in modeling presence‐only data.

## CONFLICT OF INTEREST

The authors have no conflicts of interest to declare.

## AUTHOR CONTRIBUTIONS

**Emy Guilbault:** Conceptualization (equal); Data curation (supporting); Formal analysis (lead); Methodology (equal); Software (equal); Validation (equal); Visualization (equal); Writing—original draft (lead); Writing—review and editing (equal). **Ian Renner:** Conceptualization (equal); Formal analysis (supporting); Methodology (equal); Resources (equal); Software (equal); Supervision (equal); Validation (equal); Visualization (supporting); Writing—review and editing (equal). **Michael Mahony:** Conceptualization (supporting); Data curation (equal); Resources (supporting); Supervision (supporting); Writing—review and editing (supporting). **Eric Beh:** Project administration (supporting); Supervision (supporting); Writing—review and editing (supporting).

## ETHICAL APPROVAL

This does not apply to our research.

### OPEN RESEARCH BADGES

This article has earned a Preregistered Badge for making publicly available the digitally shareable data necessary to reproduce the reported results. The data are available at https://github.com/EmyGlblt/LoopMixArticle.

## Supporting information

Appendix S1

Supplementary Material

## Data Availability

Data: The different occurrences come from the Atlas of Living Australia. The data were completed and the coordinates were verified by Michael Mahony. Observations coming from? for the same species were also added to the dataset. Data locations, the environmental covariates, and the R script to analyze the data are available at: https://doi.org/10.5061/dryad.vx0k6djqw. Rscript: An example of the scripts used for the simulation is available here: https://github.com/EmyGlblt/LoopMixArticle. A Rmarkdown document details the steps and the implementation of our functions in the same github location.

## APPENDIX A

### Testing algorithm parameters

For the parameters involved in each initialization method, we choose different values to test our model:

- knn method:$k\in \left\{1,3,5,10\right\}$
- kmeans method:$nstart\in \left\{10,15,30,50\right\}$
- LoopT method: $\delta_{\max}\in \left\{0.5,0.7,0.9\right\}$, $\delta_{\min}\in \left\{0.1,0.3,0.5,0.7\right\}$, $\delta_{\mathrm{step}}\in \left\{0.05,0.1,0.2\right\}$ where only one of the three parameters is varying at a time.
- LoopE method:$a\in \left\{1,5,10,15,20,30,40\right\}$

The results presented in Table A1 correspond to simulations with 80% of hidden observations because no major differences were found for 20% and 50% of hidden observations. The knn, kmeans, and random method did not show any differences when the parameters k and nstart, respectively, vary. All the other methods present the best performances for the parameters values displayed in Table A1.

The choices of $\delta_{\text{max}}$, $\delta_{\text{min}}$, and $\delta_{\text{step}}$ control the rate and breadth of points added to the set of locations with known species labels. As such, they control the growth of the predicted distribution as we reduce $\delta_{h}$ for each iteration h. Setting a higher value of $\delta_{\text{max}}$ such as 0.9 suggests that we first augment the distribution with only those points in which we are very confident in belonging to the species and therefore initially grow the predicted distribution slowly, while setting a lower value such as 0.5 suggests that we grow the distribution more rapidly to begin. These two values tended to be the best, with $\delta_{\text{max}}=0.5$ being optimal for test 4 with equal abundance and high correlation between distributions. The lower the value of $\delta_{\text{min}}$, the more we will grow the predicted distribution. The optimal value tends to be around 0.1, suggesting that growing the distributions significantly and therefore making use of most of the locations with unknown points is worthwhile. The value of $\delta_{\text{step}}$ controls the rate of growth between iterations. There does not seem to be a consistent winner among the three choices tested.

For the LoopE method, we increase at each iteration the number of points to add starting from a defined number $a_{1}$. While the $a_{h}$ points with highest membership probabilities are added, these membership probabilities may be small for large values of $a_{h}$, and this could explain that this method is not always doing as well as other methods. With LoopT, each species distribution grows based on equitable confidence bounds, while with LoopE, each species distribution grows based on equitable numbers of points added. The risk of LoopT is in growing the most abundant species too quickly while the risk of LoopE is in growing the least abundant species too quickly. Additionally, because we add the same numbers of points for each species, having not highly correlated species distributions may result in adding points to the wrong species. On another note, it seems that the initial number of points $a_{1}$ did not influence the performances.

### Testing species distribution for mixture methods

We present analogous results of Figures 3, 4, 5, 6 for different initialization methods.

### Estimated standard error

Here, we present the boxplots for the standard error of the predictions presented in the manuscript. The LoopT and LoopA methods tend to have lower standard error in particular at 80% of hidden observations. As expected, the individual PPM method exhibits the highest standard errors, particularly for 50% and 80% of hidden observations.

### Framework in R

We show below the steps in R to use the function for the framework presented in the manuscript. This document as well as the function used are available on github at https://github.com/EmyGlblt/LoopMixArticle.

**TABLE A1:** Summary table for parameter testing

Method	Parameter	Test 1				Test 2				Test 3				Test 4			
		MeanRSS	Acc	sumIMSE	sumcor	MeanRSS	Acc	sumIMSE	Sumcor	MeanRSS	Acc	sumIMSE	sumcor	MeanRSS	Acc	sumIMSE	sumcor
knn	k	X															
kmeans	nstart	x but only reach convergence for high starting values															
LoopT	delta max	0.5; 0.9		x		0.5; 0.9		x		0.5; 0.9		x	0.5; 0.9	0.5	0.5; 0.9	x	0.5
	delta min	0.1	x		0.1	0.1	x		0.1	0.1	x		0.1	0.1; 0.7	x		0.1
	delta step	0.1; 0.2		x		0.2		x	0.05	0.1; 0.2		x	0.05	0.1; 0.2		x	0.05, 0.1
LoopE	a	x															

Note

Tests 1, 2, 3, and 4 correspond to the test presented in the simulation part. The x sign means that there are no differences between the different values tested. The numbers displayed to represent the parameters for which the best performance is reached for each performance measure presented in the table.
