# Supplementary material for: How to make use of unlabeled observations in species distribution modeling using point process models
Source: Ecol Evol. 2021 Apr 1;11(10):5220–43. doi: 10.1002/ece3.7411 (PMC8131797; doi:10.1002/ece3.7411)

Classification of Unlabeled observations using Mixture and Loop algorithms

Emy Guilbault and Ian Renner

31 August 2020

This document runs through the different steps to use the functions ppmMixEngine and ppmLoopEngine for data classification. These functions and others are contained in `functionTestsim160420-SH.R`. First, we load the various functions and packages we will need.

```
source("functionTestsim160420-SH.r")

library(spatstat)
library(lattice)
library(latticeExtra)
library(caret)
library(viridisLite)
```

Data and environmental covariates

We load simulated data points for three species and environmental covariates store in the `PrepData.RDATA` document. We display the species true intensity as well as the three point patterns.

```
load("PrepSimData.RDATA")

# Species intensities created
Lsp1 = levelplot(sp1_int ~ X + Y, main="Sp1",
                 col.regions=colorRampPalette(c("white", "orange"))(50))
Lsp2 = levelplot(sp2_int ~ X + Y, main="Sp2",
                 col.regions=colorRampPalette(c("white", "purple"))(50))
Lsp3 = levelplot(sp3_int ~ X + Y, main="Sp3",
                 col.regions=colorRampPalette(c("white", "turquoise3"))(50))

All_pts = ppp(x=c(sp1_sim$x, sp2_sim$x, sp3_sim$x),
              y=c(sp1_sim$y, sp2_sim$y, sp3_sim$y),
              window = win, marks=c(rep("sp1", sp1_sim$n),
              rep("sp2", sp2_sim$n), rep("sp3", sp3_sim$n)))

All.plot = xyplot(All_pts$y~All_pts$x, All_pts, groups = All_pts$marks,
                  cex = 0.6, col=c("orange", "purple", "turquoise3"))

comb_levObj <- c(Lsp3, All.plot, Lsp1, Lsp2, layout=c(2,2), merge.legends = T)
update(comb_levObj, main="Species intensity distribution and point pattern")
```

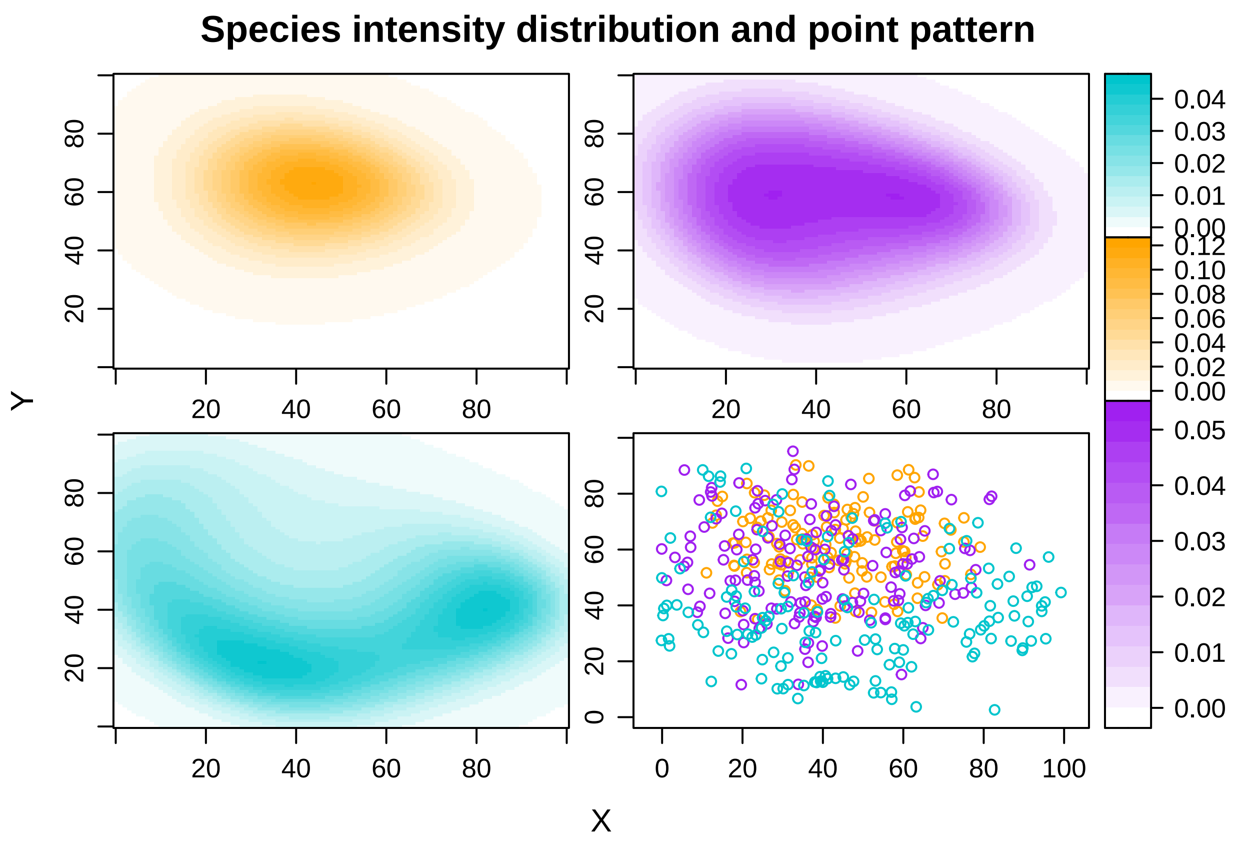

One simulation example

Mixture methods

We use a simulated dataset where we hide some label information. In the article, we choose three values and run the algorithm for all of them. Here we only present the case of 50% of hidden observations. The main function to apply that use our Mixture algorithm is ppmMixEngine, with the following arguments:

- The points with hidden observations compose the `Unknown.ppp` pattern.
- The `Known.ppp` pattern is a marked point pattern with each marks representing a known species. Both point patterns are ppp objects from the spatstat package.
- `quadsenv` is a dataframe with information on the quadrature points: coordinates x and y and environmental covariates at those points.
- `ppmform` contains the spatial trend we want the model to fit our data.
- The `initweights` argument allows to decide the method to calculate the initial weights:
- knn (we use the distance to the k nearest points). We set up the parameters to use in the initialization of the method, `k` determines the number of k nearest neighbors we want to choose for the knn nearest neighbors initialization method.
  - kmeans (we use the distance to the k centroids of the known species),
  - random (we randomly attribute initial weight to the unknown points.),
  - kps (similar to knn but the k nearest distances are calculated within each species),

  - coinF (we randomly attribute a label to the unknown points).

- We can choose the type of classification method. The argument `classif = "soft"` allows us to choose a soft classification. A hard classification is chosen using `classif = "hard"`. We apply this function to the simulated data and run both Soft and Hard classification to compare the results.

```
# models
simknn = ppmMixEngine(Known.ppp, Unknown.ppp, quadsenv = Quadmat, n.sp=3,
                      initweights = "knn", k=1, ppmform = ppmform,
                      classif = "soft")

simCF = ppmMixEngine(Known.ppp, Unknown.ppp, quadsenv = Quadmat, n.sp=3,
                     initweights = "CoinF", k=NULL, ppmform = ppmform,
                      classif = "soft")

simknn2 = ppmMixEngine(Known.ppp, Unknown.ppp, quadsenv = Quadmat, n.sp=3,
                      initweights = "knn", k=1, ppmform = ppmform,
                      classif = "hard")

simCF2 = ppmMixEngine(Known.ppp, Unknown.ppp, quadsenv = Quadmat, n.sp=3,
                      initweights = "CoinF", k=NULL, ppmform = ppmform,
                      classif = "hard")
```

Loop methods

We can do a similar job with the Loop methods. First, we run the ppmLoopEngine function where the arguments are:

- `Known.ppp`, `Unknown.ppp`, `n.sp` and `quadsenv` are the same ones used for the ppmMixEngine function.
- `addpt` allows to choose the looping method of the algorithm:
  - `"LoopA"` for all points,
  - `"LoopT"` for all points following these arguments: with membership probabilities above `delta_max` we decrease at each iteration by `delta_step` the membership probabilities till we reach `delta_min`.
  - `"LoopE"` for adding a similar number of points for each species at start and increasing by one point after the first iteration. The number of initial points to add is determined by `num.add`.

```
# models
simLoopT = ppmLoopEngine(Known.ppp, Unknown.ppp, n.sp, addpt = "LoopT",
                         quadsenv = Quadmat, ppmform= ppmform, delta_max=0.5,
                         delta_min=0.1, delta_step=0.1, num.add = NULL)

simLoopE = ppmLoopEngine(Known.ppp, Unknown.ppp, n.sp, addpt = "LoopE",
                         quadsenv = Quadmat, ppmform= ppmform, delta_max=NULL,
                         delta_min=NULL, delta_step=NULL,  num.add = 1)
```

Evaluate performance

We can access useful parameters from the algorithm run: coefficients, membership probabilities, predictions. Whether we use the Loop or the Mixture method, we can access the estimates of the covariates using the function `coef_fit`. To run the function we need the simulation object. To access the final predicted intensities and membership weights directly, we use the function `pred_int` and `member_prob` respectively. The second one need only the simulation object to run. However, for the first one, we also submit the environmental dataframe defined earlier. We also define `colpred` by a number from 1 to 3 in order to set the color of the plot from color scale available in the viridis package.

```r
# Coefficients
Coef_fit(simknn2)
```

```
##                  Sp_1       Sp_2       Sp_3
## Intercept -7.6956342  3.7436791  3.9748467
## v1         3.2153136  2.2274644 -0.9889899
## v1.2      -0.2234948 -1.5001532 -0.8037689
## v2         2.1411952  1.3145881  0.7783801
## v2.2      -0.1746103 -0.6114022 -0.9332154
```

```r
# Predictions
pred_int(simknn2, quadsenv = Quadmat, colpred="1")
```

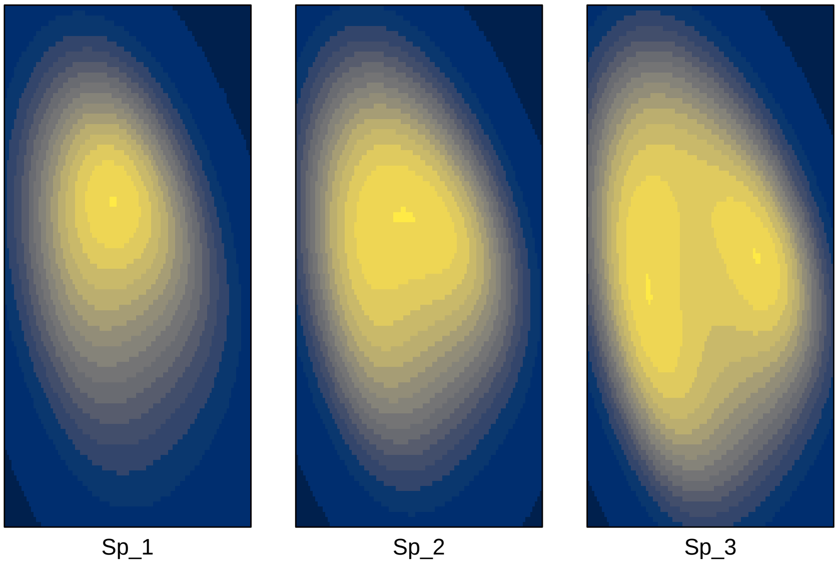


```r
# Membership probabilities
Member_prob(simknn2)
```

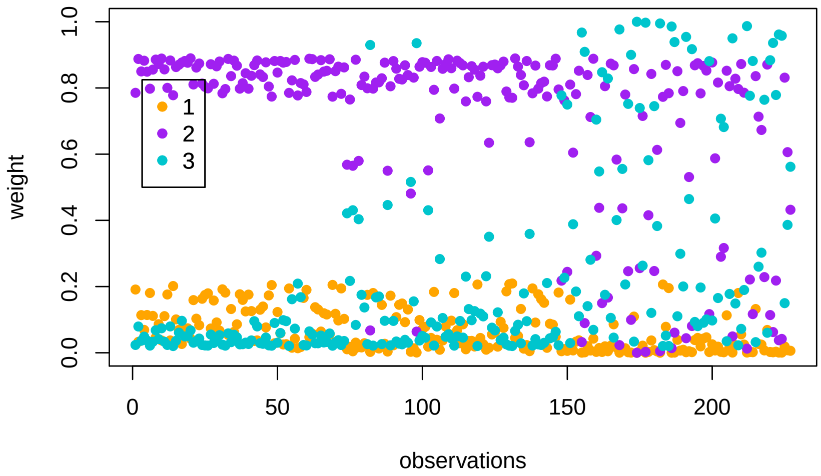

We can calculate and store the performance measures: accuracy, meanRSS, IMSE, and sumcor for each method using the Perffunc function. We need to specify:

- the fitted model object,
- the list of the true species intensities,
- the known marked point pattern defined earlier,
- the label hidden to be reclassified and the number of species.
- The pf argument helps to choose which performances we want to compute between accuracy, meanRSS, sumIMSE and sumcor. The default value NULL computes all performances.
  - For IMSE and sumcor, we can choose to compute a log or a square root of the intensity with the argument fun. By default, `fun = "Else"`, which does not modify the predicted intensity.
  - We can also decide the method to use in the calculation of the correlation (`"pearson", "kendall", "spearman"`).
- For mixture methods, the LoopM argument is set up to `FALSE` by opposition to the loop methods for which this argument will be TRUE.

```
# for performance measures
knn.perf = Perffunc(simknn, sp_int.list, n.sp=3, Known.ppp.=Known.ppp,
                    Unknown_labels.=Unknown_labels, pf = c(NULL),
                    method=c("pearson"), LoopM=FALSE)

CF.perf = Perffunc(simCF, sp_int.list, n.sp=3, Known.ppp.=Known.ppp,
                    Unknown_labels.=Unknown_labels, pf = c(NULL),
                    method=c("pearson"), LoopM=FALSE)

knn2.perf = Perffunc(simknn2, sp_int.list, n.sp=3, Known.ppp.=Known.ppp,
                    Unknown_labels.=Unknown_labels, pf = c(NULL),
                    method=c("pearson"), LoopM=FALSE)

CF2.perf = Perffunc(simCF2, sp_int.list, n.sp=3, Known.ppp.=Known.ppp,
                    Unknown_labels.=Unknown_labels, pf = c(NULL),
                    method=c("pearson"), LoopM=FALSE)


LT.perf = Perffunc(simLoopT, sp_int.list, n.sp=3, Known.ppp.=Known.ppp,
                    Unknown_labels.=Unknown_labels, pf = c(NULL),
                    method=c("pearson"), LoopM=TRUE)

LE.perf = Perffunc(simLoopE, sp_int.list, n.sp=3, Known.ppp.=Known.ppp,
                    Unknown_labels.=Unknown_labels, pf = c(NULL),
                    method=c("pearson"), LoopM=TRUE)
```

Methods comparison

We can then compare performance of the methods. If we have many simulations, we compare boxplots of the performance measures.

```
# Comparison between hard and soft classification
ACCvec2 = c(knn.perf$accmat, CF.perf$accmat, knn2.perf$accmat, CF2.perf$accmat)
meanRSSvec2 = c(knn.perf$meanRSS, CF.perf$meanRSS, knn2.perf$meanRSS, CF2.perf$meanRSS)
```

```r
IMSEvec2 = c(knn.perf$IMSE, CF.perf$IMSE, knn2.perf$IMSE, CF2.perf$IMSE)
sumcorvec2 = c(knn.perf$sumcor1, CF.perf$sumcor1, knn2.perf$sumcor1, CF2.perf$sumcor1)

Perfmixt = cbind(ACCvec2, meanRSSvec2, IMSEvec2, sumcorvec2)
rownames(Perfmixt) = c("knn", "CoinF", "knn-hard", "CoinF-hard")
Perfmixt
```

```
##              ACCvec2 meanRSSvec2  IMSEvec2 sumcorvec2
## knn        0.3127753   0.3984819 3.7693657   2.522576
## CoinF      0.3127753   0.3984814 3.7693657   2.522576
## knn-hard   0.3436123   0.4115370 0.7987194   2.859153
## CoinF-hard 0.3436123   0.4027826 0.9042866   2.876304
```

```r
# Comparison between Mixture and Loop classifcation
ACCvec = c(knn.perf$accmat, CF.perf$accmat,LT.perf$accmat, LE.perf$accmat)
meanRSSvec = c(knn.perf$meanRSS, CF.perf$meanRSS, LT.perf$meanRSS, LE.perf$meanRSS)
IMSEvec = c(knn.perf$IMSE, CF.perf$IMSE, LT.perf$IMSE, LE.perf$IMSE)
sumcorvec = c(knn.perf$sumcor1, CF.perf$sumcor1, LT.perf$sumcor1, LE.perf$sumcor1)

Perfmat = cbind(ACCvec, meanRSSvec, IMSEvec, sumcorvec)
rownames(Perfmat) = c("knn", "CoinF", "LoopT", "LoopE")
Perfmat
```

```
##          ACCvec meanRSSvec   IMSEvec sumcorvec
## knn   0.3127753  0.3984819 3.7693657  2.522576
## CoinF 0.3127753  0.3984814 3.7693657  2.522576
## LoopT 0.3127753  0.3212256 0.6698334  2.920188
## LoopE 0.3524229  0.3439102 3.8225718  2.664444
```

We can also calculate uncertainties. We choose to display and compare the prediction standard errors for species 1 only and compare the standard error map from 3 methods: knn, LoopT and indiv method.

```r
# standard error values for species 1
se.knn1 <- predict(simknn$fit.final[[1]], locations=simknn$sp_aug.list[[1]], se=TRUE)
se.CF1 <- predict(simCF$fit.final[[1]], locations=simCF$sp_aug.list[[1]], se=TRUE)
se.LT1 <- predict(simLoopT$fit.final[[1]], locations=simLoopT$sp_aug_ppp.list[[1]], se=TRUE)
se.LE1 <- predict(simLoopE$fit.final[[1]], locations=simLoopE$sp_aug_ppp.list[[1]], se=TRUE)

SEMmix.sp1 = cbind(se.knn1$se, se.CF1$se)

# boxplots
yrangeSE = range(c(min(SEMmix.sp1, se.LT1$se, se.LE1$se),
                   max(SEMmix.sp1, se.LT1$se, se.LE1$se)))

layout(matrix(c(1,1,2,3), 2, 2, byrow = TRUE))
par(mar=c(5,5,0,0)+0.1,mgp=c(4,1,0))

boxplot(se.knn1$se, se.CF1$se, se.LT1$se, se.LE1$se,
        col = c("yellow","yellow","green","green","green"),
        names = c("knn", "CF", "LoopT", "LoopE"), at = c(1,2, 4,5),
        ylab = "Se for sp1", ylim=yrangeSE, las = 2, cex=1.3, cex.lab=1.3, cex.axis=1.3)

# standard error plots
seplot.knn <- predict(simknn$fit.final[[1]], se=TRUE)
```

```r
seplot.LT <- predict(simLoopT$fit.final[[1]], se=TRUE)

Xplot = as.data.frame(simknn$fitaft.pred[[1]])$x
Yplot = as.data.frame(simknn$fitaft.pred[[1]])$y

print(levelplot(as.vector(seplot.knn$se$v) ~ Xplot + Yplot, col.regions=viridis(20),
                main="knn method standard error", ylab=NULL, xlab=NULL),
      split=c(1, 2, 2, 2), newpage=FALSE)
print(levelplot(as.vector(seplot.LT$se$v) ~ Xplot + Yplot, col.regions=viridis(20),
                main="LoopT method standard error", ylab=NULL, xlab=NULL),
      split=c(2, 2, 2, 2), newpage=FALSE)
```

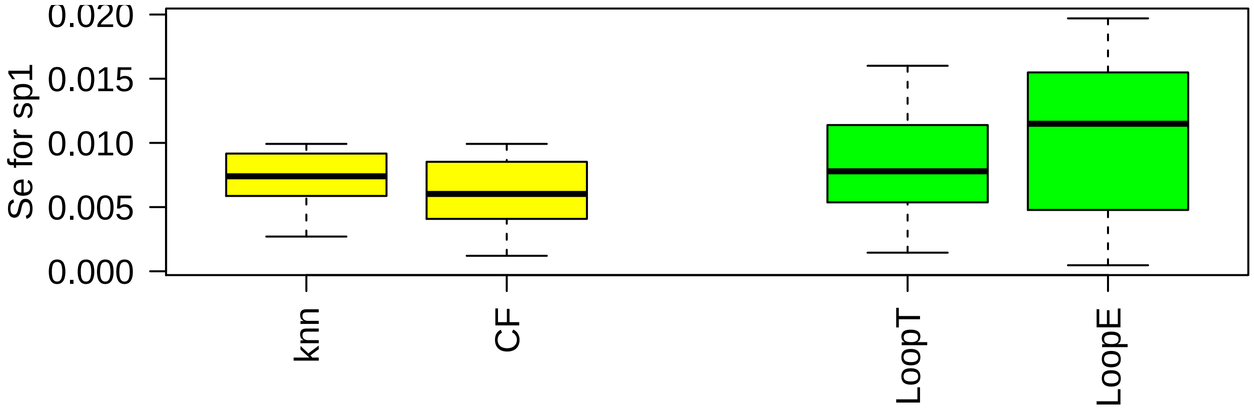

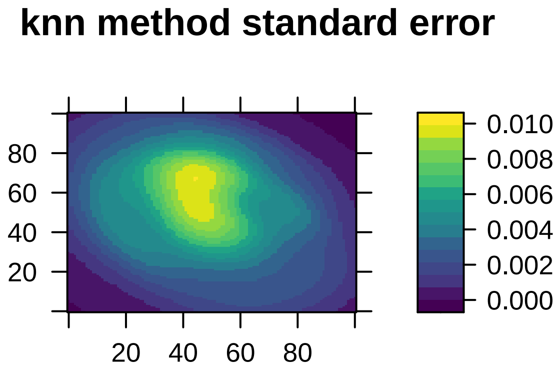

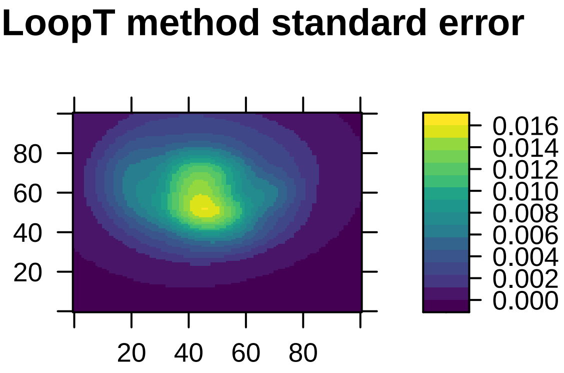

Supplement: Supplementary file 2 — Supplementary Material [file ECE3-11-5220-s001.pdf]
